# Effect of *Akkermansia muciniphila* on GLP-1 and Insulin Secretion

**DOI:** 10.3390/nu17152516

**Published:** 2025-07-31

**Authors:** Ananta Prasad Arukha, Subhendu Nayak, Durga Madhab Swain

**Affiliations:** 1 Department of Infectious Disease and Immunology, University of Florida, Gainesville, FL 32608, USA; ananta.arukha@ufl.edu; 2 Vidya USA Corporation, 7 Otis Stone Hunter Road, Bunnell, FL 32100, USA; subhendu@vidyaherbsusa.com

**Keywords:** *Akkermansia muciniphila*, diabetes, insulin secretion, probiotics, GLP-1 secretion

## Abstract

**Background/Objectives:** Gut microbiota research has gained momentum in recent years broadening knowledge of microbial components and their potential effects on health and well-being. Strong association between explicit microbes and metabolic diseases associated with obesity and type 2 diabetes mellitus, gastrointestinal disorders, neurodegenerative diseases, and even cancers have been established. *Akkermansia muciniphila* is a budding next-generation probiotic that plays an important role in systemic metabolism, intestinal health, and immune regulation, establishing strong implications for its use as a potent therapeutic intervention in diverse diseases. This project aimed at evaluating whether bacterial cell extracts of VH *Akkermansia muciniphila* (Vidya Strain; VS) can stimulate insulin secretion in INS-1 pancreatic beta cells and GLP-1 secretion in NCI-H716 human L-cells, both established in vitro models for studying metabolic regulation. **Methods:** Cultured VH *Akkermansia muciniphila* extracts were administered in a dose-dependent manner on INS-1 cells, and glucose-stimulated insulin secretion (GSIS) was measured via ELISA. Treated Human L-cell lines (NCI-H716) were analyzed for GLP-1 secretion. **Results:** Our study demonstrated that VH *Akkermansia muciniphila* extracts modestly increase insulin secretion from INS-1 beta cells and, more notably, induce a robust, dose-dependent rise in GLP-1 secretion from NCI-H716 L-cells, with the highest dose achieving over a 2000% increase comparable to glutamine. **Conclusions:** These findings suggest that VH *A. muciniphila* extracts may offer metabolic benefits by enhancing GLP-1 release, highlighting their potential for managing type 2 diabetes and obesity.

## 1. Introduction

Diabetes mellitus is a chronic metabolic disease demarcated by elevated blood glucose levels due to impaired insulin secretion, insulin action, and at times both. Persistent hyperglycemia often leads to serious complications that affect both small and large blood vessels. Diabetes is a complication that is globally rising, with the greatest increase observed in low- and middle-income countries, especially as urbanization and lifestyle changes accelerate [1]. The International Diabetes Federation estimates that the number of adults affected by diabetes may rise to 589 million globally. About 11.1% of the population aged 20–79 is living with diabetes, and over 40% are still undiagnosed. Projections indicate that if current trends continue, the number of people with diabetes could reach 853 million by 2050. Rapid urbanization and shifts in dietary patterns have contributed to a particularly high and growing prevalence in these regions. These figures underscore the urgent need for effective prevention, early detection, and management strategies to address the increasing global burden of diabetes.

A critical angle of diabetes is the dysbiosis in the existence of insulin, which is a peptide hormone produced by pancreatic β-cells that plays a central role in maintaining glucose homeostasis by promoting glucose uptake into skeletal muscle and adipose tissue, stimulating glycogen synthesis in the liver and suppressing hepatic glucose production [2]. Beyond its effects on carbohydrate metabolism, insulin also facilitates lipid storage, inhibits lipolysis, and enhances protein synthesis, thereby supporting overall metabolic health [3]. The disruption of insulin secretion, whether due to autoimmune destruction of β-cells in type 1 diabetes or insulin resistance in peripheral tissues as seen in type 2 diabetes, is fundamental to the pathogenesis of diabetes and its associated complications [4].

As the global prevalence of diabetes continues to rise, largely driven by increasing rates of obesity, sedentary lifestyles, and poor dietary habits, the effective management of insulin secretion and sensitivity becomes imperative for a healthy life. Therefore, the development of therapies that enhance insulin action remains a major focus of research.

Yet another key player in diabetes dysbiosis is the gut hormone glucagon-like peptide-1 (GLP-1), which is secreted by intestinal L-cells. GLP-1 is a critical regulator of glucose homeostasis and appetite [5,6]. It stimulates glucose-dependent insulin secretion, suppresses glucagon release, and delays gastric emptying, making it a cornerstone in managing type 2 diabetes and obesity. GLP-1 secretion is modulated by nutrient sensing in L-cells, particularly through protein-rich foods, unsaturated fatty acids, and soluble fiber, which enhance its release. Furthermore, GLP-1 receptor agonists (GLP-1 RAs) have demonstrated significant clinical benefits, including sustained weight loss (10–20% body weight reduction) and cardiovascular risk reduction through mechanisms such as improved endothelial function, reduced inflammation, and the attenuation of atherosclerotic plaque progression [7]. Recent studies highlight their potential neuroprotective effects, with a 10–20% lower risk of Alzheimer’s disease and dementia, likely due to anti-inflammatory actions and the modulation of brain reward pathways.

The gut is revered as the second brain. Scientific studies have established the significant influence of the gut microbiome on insulin secretion and sensitivity. Certain gut bacteria produce metabolites such as short-chain fatty acids (SCFAs) like butyrate and acetate, which improve insulin sensitivity and promote insulin secretion, partly by modulating the inflammatory pathways implicated in insulin resistance. This insinuates that targeting the gut microbiota with probiotics or postbiotic metabolites may offer promising, natural strategies for improving insulin function and managing diabetes and related metabolic disorders.

*Akkermansia muciniphila*, a keystone gut microbe, enhances host health through unique mucin degradation [8] and metabolic activities. Its distinctive capability to break down intestinal mucin glycoproteins occurs via glycosyl hydrolases, sulfatases, and sialidases-generates short-chain fatty acids (SCFAs) like acetate and propionate [9]. These SCFAs serve as energy sources for colonocytes, promote regulatory T cell differentiation, and suppress inflammation by inhibiting histone deacetylases (HDACs) [8,9].

In this regard, we endeavored to conduct clinical studies to evaluate whether the bacterial cell extracts of the probiotic *Akkermansia muciniphila* can stimulate insulin secretion in pancreatic beta cells (using the INS-1 cell line) and GLP-1 secretion in human L-cells (using the NCI-H716 cell line). Both cell lines are well-established models for evaluating GLP-1 and insulin secretion, respectively, and for screening compounds that modulate these hormones. By surveying the effects of VH (Vidya Herbs) *A. muciniphila* extracts on hormone secretion in these cell lines, the feasibility of exploring their potential as metabolic interventions for improving glucose regulation and supporting diabetes and weight management increases exponentially.

## 2. Materials and Methods

### 2.1. Bacterial Cell Preparation and Protein Quantification

VH *Akkermansia muciniphila* cells were cultured under anaerobic conditions to obtain sufficient biomass at the Vidya Herbs sterile facility. Bacterial cells were lysed via ultra-strength sonication (intensity, 40% for a duration of 10 min, 2 cycles were performed (5 min each cycle) having 10 s on and 10 s off for a total of 2 cycles), applying high-intensity sound waves to disrupt cell walls. The lysate was centrifuged to pellet insoluble debris, and the soluble protein-rich supernatant was collected. Protein quantification was performed using a bicinchoninic acid (BCA) assay.

#### 2.1.1. Insulin Study

Treatment Formulation

A dose-response experiment was designed with protein concentrations ranging from 0 μg/mL (negative control) to 500 μg/mL of total protein. INS-1 cells were treated with these formulated doses, and glucose-stimulated insulin secretion (GSIS) was measured via ELISA at 0, 30, and 60 min to assess time-dependent effects. This protocol ensured the precise evaluation of VH *A. muciniphila*-derived proteins on insulin secretion dynamics.

Cell Line Preparation, Culture Conditions, and Insulin Measurement

INS-1 cells were cultured in RPMI-1640 medium supplemented with 10% fetal bovine serum (FBS) and maintained at 37 °C in a humidified incubator with 5% CO_2_. Cells were seeded into 12-well plates and allowed to reach confluency prior to treatment, with all assays performed in biological triplicate. The two controls included were negative control consisting of untreated cells (0 µg/mL extract) and a positive control with cells exposed to 10 mM glucose, a known stimulator of insulin secretion in pancreatic beta cells. Following treatment, cell culture supernatants were collected at 0, 30, and 60 min, and insulin concentrations were quantified using a commercially available enzyme-linked immunosorbent assay (ELISA). All insulin measurements were performed in triplicate to ensure the accuracy and reproducibility of results.

#### 2.1.2. GLP-1 Study

Treatment Formulation

A dose-response experiment was designed with protein concentrations ranging from 0 μg/mL (negative control) to 500 μg/mL of total protein. The treatment regimen involved exposing the NCI-H716 cells to these concentrations and the measurement of GLP-1 secretion at three time points: 0, 30, and 60 min.

Cell Line Preparation, Culture Conditions, and GLP-1 Measurement

Human L-cell lines (NCI-H716) were cultured in RPMI-1640 medium supplemented with 10% fetal bovine serum (FBS) and maintained under standard conditions (37 °C, 5% CO_2_). Cells were seeded into 12-well plates at a density that allowed them to reach confluency before the treatment. Two controls were included in the experiment: a negative control, cells without any treatment (0 µg/mL extract); and a positive control, cells treated with 10 mM glutamine, known to stimulate GLP-1 secretion. After the treatment, cell culture supernatants were collected at 0, 30, and 60 min and analyzed for GLP-1 secretion using a commercially available enzyme-linked immunosorbent assay (ELISA). GLP-1 concentrations were measured in triplicate.

### 2.2. Data Analysis

Data was analyzed using standard statistical software, including Excel and GraphPad Prism Version 9, and expressed as means ± standard deviations (SDs) and standard errors of the mean (SEMs). Statistical significance between groups was evaluated using one-way ANOVA followed by Tukey’s post-hoc test for multiple comparisons.

## 3. Results

**Figure 1:**
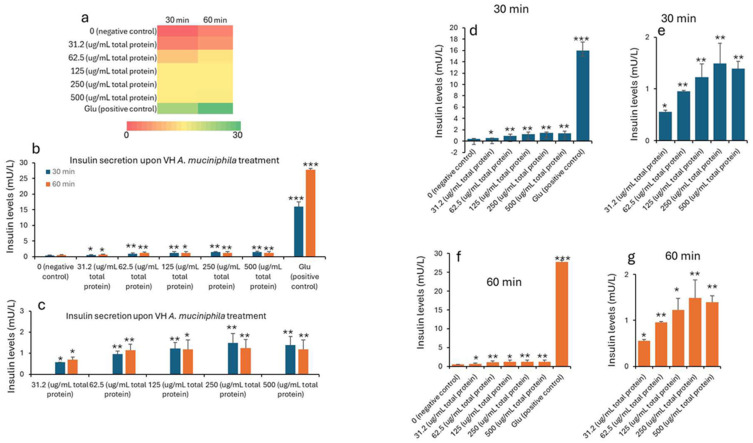
Insulin levels (mU/L) in INS1 cells treated with VH *Akkermansia muciniphila* cell extract at various doses (0 [negative control], 31.2, 62.5, 125, 250, and 500 µg/mL total protein) at 0 min (baseline) and after 30 and 60 min of treatment. (**a**) Heatmap depicting insulin levels (red indicates low, yellow indicates medium, and green indicates high levels) and secretion. (**b**,**d**,**f**) Insulin levels following different doses of VH *Akkermansia muciniphila* treatment at both 30 and 60 min (**b**), and at 30 min (**d**) and 60 min (**f**) time points. (**c**,**e**,**g**) Insulin levels in groups only treated with 31.2, 62.5, 125, 250, and 500 µg/mL total protein after 30 and 60 min of treatments. All assays were performed with three biological replicates. Values presented in bars are means, and error bars indicate SD. *p*-Values indicated with *** < 0.001 are statistically significantly different compared to the negative control. * = *p* < 0.05 (statistically significant) and ** = *p* < 0.01 (more statistically significant).

**Figure 2:**
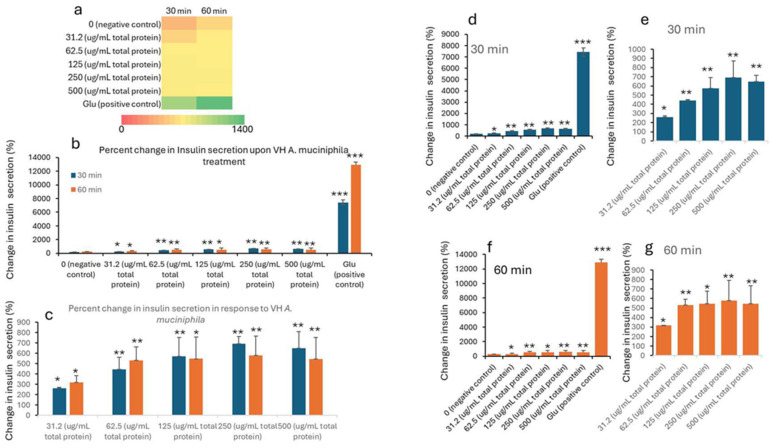
Percent change in insulin levels (mU/L) in INS1 cells treated with VH *Akkermansia muciniphila* cell extract at various doses (0 [negative control], 31.2, 62.5, 125, 250, and 500 µg/mL total protein) at 0 min (baseline) and after 30 and 60 min of treatment. (**a**) Heatmap depicting insulin levels (red indicates low, yellow indicates medium, and green indicates high change) and secretion. (**b**,**d**,**f**) Percent changes in insulin levels following different doses of VH *Akkermansia muciniphila* treatment at both 30 and 60 min (**b**), and at 30 min (**d**) and 60 min (**f**) time points. (**c**,**e**,**g**) Insulin levels in groups only treated with 31.2, 62.5, 125, 250, and 500 µg/mL total protein after 30 and 60 min of treatments. All assays were performed with three biological replicates. Values presented in bars are means, and error bars indicate SD. *p*-Values indicated with *** < 0.001 are statistically significantly different compared to the negative control. * = *p* < 0.05 (statistically significant) and ** = *p* < 0.01 (more statistically significant).

Insulin concentrations were seen to improve with the treatment. The data in Table 1 and Table 2 shows the treatment of INS-1 beta cells with increasing concentrations of VH *A. muciniphila* cell extracts affects insulin secretion over time, compared to negative (untreated) and positive (glucose-stimulated) controls. At baseline (0 min), insulin levels are low and similar across all groups, indicating no immediate effect from the extract (Figure 1). After 30 and 60 min of treatment, there is a clear, dose-dependent increase in insulin secretion. Higher concentrations of the VH *A. muciniphila* extract result in significantly greater insulin release. This is visually summarized in the (Figure 1) heatmap (panel a), where the color shifts from red (low) to green (high) as both the dose and time increase. After 30 and 60 min, insulin secretion increases in a dose-dependent manner with higher concentrations of VH *A. muciniphila* extract with an optimum at the extract dose of 250 µg/mL. The positive control (10 mM glucose) produces a much larger increase in insulin secretion (15.9 mU/L at 30 min, 27.7 mU/L at 60 min), consistent with the robust glucose-stimulated response expected in INS-1 cells. When focusing only on the treated groups (Table 2, Figure 2), the data reinforces that even at the lowest tested dose (31.2 µg/mL), insulin secretion is significantly higher than the negative control after 30 and 60 min. Overall, the results indicate that VH *A. muciniphila* cell extracts stimulate insulin secretion in a dose-dependent manner, though to a lesser extent than direct glucose stimulation, supporting their potential to modulate beta-cell function.

**Figure 3:**
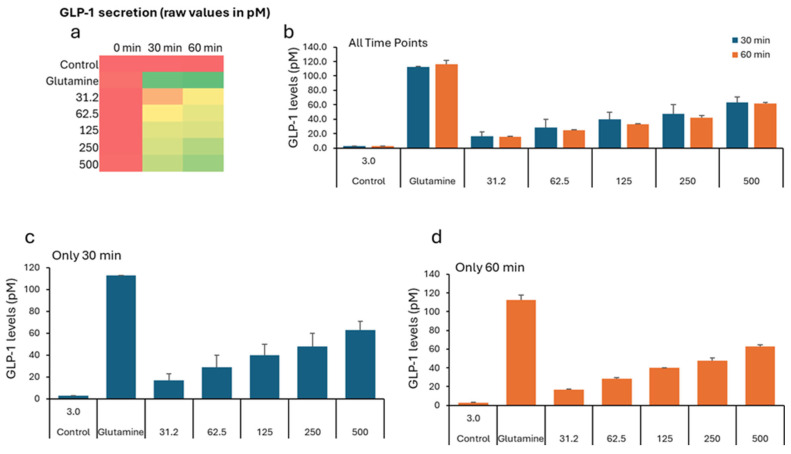
GLP-1 levels (pM) in NCI-H716 cells treated with VH *Akkermansia muciniphila* cell extract at various doses (0 [negative control], 31.2, 62.5, 125, 250, and 500 µg/mL total protein) at 0 min (baseline) and after 30 and 60 min of treatment. (**a**) Heatmap depicting GLP-1 levels (red indicates low, yellow indicates medium, and green indicates high levels) and secretion. (**b**–**d**) GLP-1 levels following different doses of VH *Akkermansia muciniphila* treatment at both 30 and 60 min (**b**), and at 30 min (**c**) and 60 min (**d**) time points. All assays were performed in triplicate. Values presented in bars are means, and error bars indicate SD.

**Figure 4:**
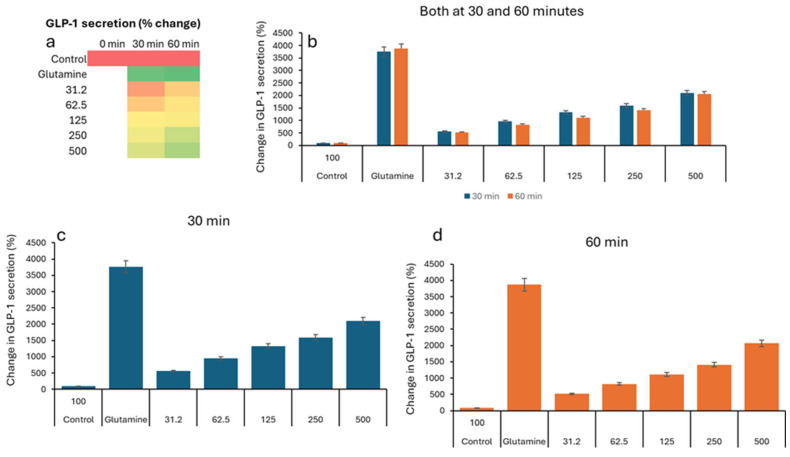
Percent change in GLP-1 secretion relative to the negative control, in NCI-H716 cells treated with VH *Akkermansia muciniphila* cell extract at various doses (0 [negative control], 31.2, 62.5, 125, 250, and 500 µg/mL total protein) at 0 min (baseline) and after 30 and 60 min of treatment. (**a**) Heatmap depicting change in GLP-1 levels (red indicates low, yellow indicates medium, and green indicates high levels) and secretion. (**b**–**d**) Change in GLP-1 levels following different doses of VH *Akkermansia muciniphila* treatment at both 30 and 60 min (**b**), and at 30 min (**c**) and 60 min (**d**) time points. All assays were performed in triplicate. Values presented in bars are means, and error bars indicate SD.

In the GLP study, all groups show low GLP-1 levels (2–5 pM), indicating similar starting points before treatment (Table 3). The negative control remains low (2.76 pM). All VH *Akkermansia muciniphila* extract-treated groups show a marked, dose-dependent increase in GLP-1 secretion. Figure 3a (Heatmap) shows a clear transition from low (red) to high (green) GLP-1 levels with an increasing extract dose and time. While the Figure 1b–d (bar graphs) illustrates the dose- and time-dependent increase in GLP-1 secretion, with the highest levels at 60 min and 500 µg/mL, Figure 4 illustrates the percent change in GLP-1 secretion from NCI-H716 cells after treatment with various doses of VH *Akkermansia muciniphila* cell extract, compared to a negative control and a positive control (glutamine), measured at 0, 30, and 60 min. The heatmap shows a clear progression from red, low GLP-1 secretion at baseline, to yellow and green, higher secretion, as both the extract dose and incubation time increase. Error bars (SD) are relatively small, indicating consistent results across replicates. The VH *Akkermansia muciniphila* cell extract significantly increases GLP-1 secretion from NCI-H716 cells in a dose- and time-dependent manner, with effects that approach those of the positive control (glutamine) at higher doses and longer incubation. This supports the potential of VH *Akkermansia muciniphila* as a beneficial modulator of gut hormone secretion and metabolic health.

## 4. Discussion

Chronic inflammation in type 2 diabetes mellitus (T2DM) is closely linked to dysregulated insulin secretion including insulin resistance and disrupted immune responses. The lipopolysaccharide (LPS) also plays a key role in driving low-grade inflammation, likely due to impaired clearance mechanisms and gut-derived endotoxin absorption. Studies suggest a bidirectional relationship between metabolic dysfunction and innate immune activation [10,11]. Hence, an ideal therapeutic candidate for diabetes treatment and management should aid in metabolic regulation, significantly impact insulin secretion and sensitivity and appetite regulation, and reduce immune system inflammation.

Gut bacteria influence diabetes through immune and metabolic pathways. High lipopolysaccharide (LPS) levels in type 2 diabetes mellitus (T2DM) patients activates the Toll-like receptor 4 (TLR4), which triggers macrophage recruitment and NF-κB signaling [12]. These promote systemic inflammation and suppress insulin secretion by disrupting the β-cell function [13]. This inflammatory cascade exacerbates insulin resistance and hyperglycemia. However, the beneficial gut microbes can counter these effects by metabolizing primary bile acids into secondary bile acids, which activate the farnesoid X receptor (FXR), stimulating fibroblast growth factor 19/15 (FGF19/15) release. This enhances insulin sensitivity and glucose tolerance. Among the beneficial gut bacteria strains, *Akkermansia muciniphila*, a key probiotic, has been established to strengthen the intestinal barrier integrity and reduce LPS translocation and inflammation, while promoting GLP-1 secretion to improve glycemic control [13].

Our study demonstrates that VH *Akkermansia muciniphila* extracts modestly stimulate insulin secretion from INS-1 pancreatic beta cells in a dose-dependent manner. While these increases are statistically significant, they remain substantially lower than those induced by classical insulin secretagogues such as glucose. However, the study suggests that VH *Akkermansia muciniphila* harbors bioactive molecules that are capable of enhancing insulin production.

In parallel, our study also reveals a robust, dose-dependent increase in GLP-1 secretion from NCI-H716 gut L-cells upon exposure to VH *A. muciniphila* cell extracts. At the highest tested concentration (500 μg/mL), GLP-1 secretion increased by over 2000% compared to the negative control, reaching levels comparable to glutamine, a well-characterized GLP-1 secretagogue. GLP-1 is a pivotal incretin hormone that enhances insulin secretion, making it a key target in the management of type 2 diabetes and obesity [14]. The ability of *A. muciniphila* extracts to stimulate GLP-1 release suggests a novel mechanism by which this bacterium may exert metabolic benefits.

The therapeutic implications are noteworthy, particularly for metabolic disorders such as type 2 diabetes. Enhancing endogenous insulin secretion could contribute to improved glycemic control and potentially reduce the burden of exogenous insulin therapy. Several studies have highlighted the beneficial metabolic effects of *A. muciniphila*, including improved insulin sensitivity and glucose homeostasis in both animal models and human subjects [15,16]. The ability of these extracts to enhance insulin release from INS-1 cells, although less potent than classical secretagogues such as glucose, suggests the presence of bioactive components that may support endogenous insulin production. This finding is particularly relevant for the development of novel interventions aimed at improving glycemic control in individuals with diabetes, a concept supported by recent studies demonstrating the metabolic benefits of *A. muciniphila* supplementation in both preclinical and clinical settings [15,17].

Furthermore, the robust stimulation of GLP-1 secretion observed in this study highlights an additional mechanism by which VH *A. muciniphila* may promote metabolic health. Recent research has demonstrated that *Akkermansia muciniphila* produces bioactive components capable of stimulating GLP-1 secretion from intestinal L-cells, crucial for metabolic regulation, including insulin secretion, appetite control, and gastric motility. Enhancing GLP-1 levels through dietary or probiotic strategies may offer a promising approach for managing metabolic diseases such as type 2 diabetes, obesity, and cardiovascular conditions.

Specifically, the secreted protein P9 from *A. muciniphila* has been identified as a key factor that binds to intercellular adhesion molecule 2 (ICAM-2) on L-cells, directly triggering GLP-1 release and improving glucose homeostasis in animal models [18]. While this protein is a significant mediator, *A. muciniphila* also produces a variety of other metabolites, proteins, and cell wall components that may contribute to its effects on GLP-1 secretion, though the roles of these additional factors are still being investigated.

Research also supports the role of *A. muciniphila* and its metabolites in modulating gut hormone secretion and metabolic health. Ref. [19] found that *A. muciniphila*-derived extracellular vesicles (AmEVs) enhance intestinal barrier integrity by upregulating tight junction proteins such as occludin through AMPK activation, reducing gut permeability, and improving glucose tolerance in murine models. Additionally, ref. [17] showed that both pasteurized *A. muciniphila* and its outer membrane protein Amuc_1100 reduced adiposity and insulin resistance in mice to improve glucose tolerance and increase GLP-1 levels in animal models by strengthening the gut barrier function and activating TLR2 signaling [17]. The bacterium produces various bioactive components, including outer membrane proteins, short-chain fatty acids, and extracellular vesicles, that may interact with intestinal L-cells to promote GLP-1 secretion [20]. *A. muciniphila*-derived short-chain fatty acids (SCFAs), such as propionate, enhance GLP-1 release through free fatty acid receptor (FFAR) activation, further supporting glycemic control [13].

Metformin, an antidiabetic agent, was found to increase the abundance of *A. muciniphila* in high-fat-diet-fed mice, correlating with improved glycemic control and reduced adipose inflammation through T-regulatory cell recruitment. In humans, metformin-responsive T2DM patients exhibit higher fecal *A. muciniphila* levels compared to non-responders.

In diabetic mouse models, *A. muciniphila* supplementation mitigated hepatic dysfunction, oxidative stress, and inflammation. Vancomycin-treated NOD mice with elevated *A. muciniphila* levels showed a reduced T1DM incidence, underscoring its protective role. A 12-week clinical trial by [17] using the probiotic blend WBF-011 (containing *A. muciniphila*) significantly improved postprandial glucose and HbA1c in T2DM patients, likely via butyrate-induced GLP-1 release.

The increasing popularity of *A. muciniphila* as a metabolic therapeutic for diabetes can be gauged from the ongoing clinical trials NCT04797442 and NCT05114018. By enhancing insulin secretion, *A. muciniphila* could help regulate blood glucose levels more effectively, offering a natural approach to diabetes and related disorder management.

It is important to note that the findings of our study are based on in vitro models, which may not fully recapitulate the complexity of human physiology. In vivo studies in animal models and clinical trials in humans are necessary to confirm the physiological relevance, safety, and efficacy of *A. muciniphila* extracts for metabolic health interventions. Such studies should also investigate the long-term effects of modulating GLP-1 and insulin secretion, including impacts on weight management, glucose homeostasis, and cardiovascular risk.

With the global burden of metabolic diseases surpassing an estimated 537 million people living with diabetes in 2021 based on the International Diabetes Federation estimate, a safe, cost-effective, and multi-targeted therapeutic approach is imperative for overall health management.

## 5. Conclusions

This investigation provides compelling evidence that the cell extracts of the VH *Akkermansia muciniphila* strain exert beneficial effects on key metabolic pathways by stimulating both insulin secretion from pancreatic beta cells and GLP-1 secretion from human L-cells in a dose-dependent manner. As a probiotic strain that regulates gut homeostasis, undeniably, VH *A. muciniphila* represents a promising avenue for metabolic health interventions, with dual actions on insulin and GLP-1 secretion. To completely comprehend their true therapeutic potential and long-term usage safety, further studies on isolating and characterizing the active components, elucidating their mechanisms of action, and evaluating their clinical utility for the prevention and management of metabolic diseases such as type 2 diabetes and obesity are warranted.

## Figures and Tables

**Table 1:** Insulin concentration (mU/L) levels in media supernatant before and after treatment of INS-1 cells with VH *A. muciniphila* cell extracts.

Treatment (µg/mL) *	0 min (Baseline)			30 min				60 min			
	Mean	SD	SEM	Mean	SD	SEM	*p* Values	Mean	SD	SEM	*p* Values
0 (negative control)	0.25	0.20	0.07	0.41	0.03	0.01		0.57	0.01	0.00	
31.2 (μg/mL total protein)	0.18	0.06	0.02	0.56	0.02	0.01	0.027	0.68	0.14	0.05	0.0182
62.5 (μg/mL total protein)				0.95	0.25	0.08	0.009	1.14	0.29	0.10	0.0057
125 (μg/mL total protein)				1.23	0.39	0.13	0.008	1.18	0.45	0.15	0.0158
250 (μg/mL total protein)				1.49	0.14	0.05	0.0004	1.25	0.40	0.13	0.0092
500 (μg/mL total protein)				1.39	0.35	0.12	0.0039	1.17	0.45	0.15	0.0153
Glu (positive control)				15.9	1.51	0.50	2.8 × 10^−5^	27.7	0.45	0.15	4.08 × 10^−7^

* The total protein concentrations of 31.2, 62.5, 125, 250, and 500 μg/mL are equivalent to 0.625, 1.25, 2.5, 5, and 10 billion CFUs per well, respectively. All the assays were performed with three biological replicates.

**Table 2:** Percent change in insulin secretion in INS-1 cells after treatment with *A. muciniphila* cell extracts.

Treatment (µg/mL) *	0 min (Baseline)			30 min				60 min			
	Mean	SD	SEM	Mean	SD	SEM	*p* Values	Mean	SD	SEM	*p* Values
0 (negative control)	117.42	91.57	30.52	191.34	13.52	4.51		263.71	2.48	0.83	
31.2 (μg/mL total protein)	82.58	27.84	9.28	260.39	8.51	2.84	0.027	317.28	64.06	21.35	0.0182
62.5 (μg/mL total protein)				443.78	116.81	38.94	0.009	530.95	132.97	44.32	0.0057
125 (μg/mL total protein)				572.27	180.74	60.25	0.008	546.95	210.22	70.07	0.0158
250 (μg/mL total protein)				693.30	67.18	22.39	0.0004	579.84	187.53	62.51	0.0092
500 (μg/mL total protein)				647.64	162.44	54.15	0.0039	546.33	207.60	69.20	0.0153
Glu (positive control)				7434.4	701.2	233.7	2.8 × 10^−5^	12,898.6	415.49	138.5	4.08 × 10^−7^

* The total protein concentrations of 31.2, 62.5, 125, 250, and 500 μg/mL are equivalent to 0.625, 1.25, 2.5, 5, and 10 billion CFUs per well, respectively. All the assays were performed with three biological replicates.

**Table 3:** Concentration of GLP-1 (pM) levels in media supernatant before and after treatment with VH *A. muciniphila* cell extracts and glutamine as a positive control.

Treatment (µg/mL) *	0 min (Baseline)			30 min			60 min		
	Mean	SD	SEM	Mean	SD	SEM	Mean	SD	SEM
0 (Negative Control)	2.82	0.89	0.30	2.76	0.25	0.08	2.59	0.46	0.15
31.2	2.42	0.35	0.12	25.78	3.45	1.15	45.66	4.74	1.58
62.5	2.32	0.20	0.07	43.79	6.85	2.28	54.93	10.85	3.62
125	2.49	0.46	0.15	58.03	11.26	3.75	60.53	9.22	3.07
250	2.40	0.17	0.06	62.92	2.79	0.93	78.46	11.77	3.92
500	3.45	0.35	0.12	72.10	7.74	2.58	89.57	8.58	2.86
Positive Control (Glutamine-10 mM)	5.07	2.21	0.74	112.63	0.48	0.16	115.93	5.23	1.74

* The total protein concentrations of 31.2, 62.5, 125, 250, and 500 μg/mL are equivalent of 0.625, 1.25, 2.5, 5, and 10 billion/mL CFUs, respectively.

## Data Availability

The original contributions presented in this study are included in the article. Further inquiries can be directed to the corresponding author.
